# Dispersive Solid-Phase Extraction Using Magnetic Carbon Nanotube Composite for the Determination of Emergent Mycotoxins in Urine Samples

**DOI:** 10.3390/toxins12010051

**Published:** 2020-01-15

**Authors:** Natalia Arroyo-Manzanares, Rosa Peñalver-Soler, Natalia Campillo, Pilar Viñas

**Affiliations:** Department of Analytical Chemistry, Faculty of Chemistry, Regional Campus of International Excellence “Campus Mare Nostrum”, University of Murcia, E-30100 Murcia, Spain; natalia.arroyo@um.es (N.A.-M.); rosamaria.penalver@um.es (R.P.-S.); ncampi@um.es (N.C.)

**Keywords:** emergent mycotoxins, urine, dispersive solid-phase extraction, magnetic carbon nanotube composite

## Abstract

Dispersive magnetic solid-phase extraction (DMSPE) has received growing attention for sample treatment preconcentration prior to the separation of analytes due to its many advantages. In the present work, the potential of DMSPE for the determination of emergent mycotoxins (enniatins A, A1, B and B1, and beauvericin) is investigated for the first time. Different magnetic nanoparticles were tested and a magnetic multiwalled carbon nanotube (Fe_3_O_4_@MWCNT) composite was selected for the extraction and preconcentration of the five target mycotoxins in human urine samples before their analysis by ultrahigh performance liquid chromatography coupled to high resolution mass spectrometry (UHPLC-HRMS). The nanocomposite was characterized by energy dispersive X-ray spectrometry, scanning electron microscopy, Fourier transform infrared spectrophotometry, and X-ray diffraction. Several parameters affecting the adsorption and desorption of DMSPE steps were optimized and the method was fully validated. Due to a matrix effect, matrix-matched calibration curves were necessary to carry out quantification. In this way, limits of quantification of between 0.04 and 0.1 μg/L, relative standard deviation values lower than 12% and recoveries between 89.3% and 98.9% were obtained. Finally, a study of the reuse of the Fe_3_O_4_@MWCNT composite was carried out, confirming that it can be reused at least four times.

## 1. Introduction

Mycotoxins are low molecular weight noxious secondary metabolites produced by toxicogenic strains of some mould species that mainly belong to the genera *Aspergillus*, *Fusarium*, *Penicillium*, *Alternaria*, and *Claviceps*. To date, approximately 400 different mycotoxins have been described, although the most common are aflatoxins (B_1_, B_2_, G_1_, and G_2_), ochratoxin A, fumonisins, trichothecenes, zearalenone, and patulin. Mycotoxins can contaminate animal feed, food, or the raw materials used for their production, originating diseases and disorders, both in humans and animals. Mycotoxins can bioaccumulate in fluids, tissues and organs, especially the liver and kidney, as well as affect the nervous, endocrine, and immune systems. Due to their toxicity, the European Union has established or recommended maximum permissible contents for some of these contaminants in various foods [1,2,3]. 

Among mycotoxins, enniatins and beauvericins have aroused great interest in recent years. These compounds, also known as emerging mycotoxins, due to their recent discovery, are produced by *Fusarium* species and, although no specific legislation has dealt with them yet, their toxicity-including genotoxicity, cytotoxicity, and effects on the reproductive system, has been demonstrated in in vitro studies [4]. The European Food Safety Authority (EFSA) suggests that chronic exposure to these mycotoxins might be a concern and should be confirmed with more acute toxicological data [5].

**Figure 1:**
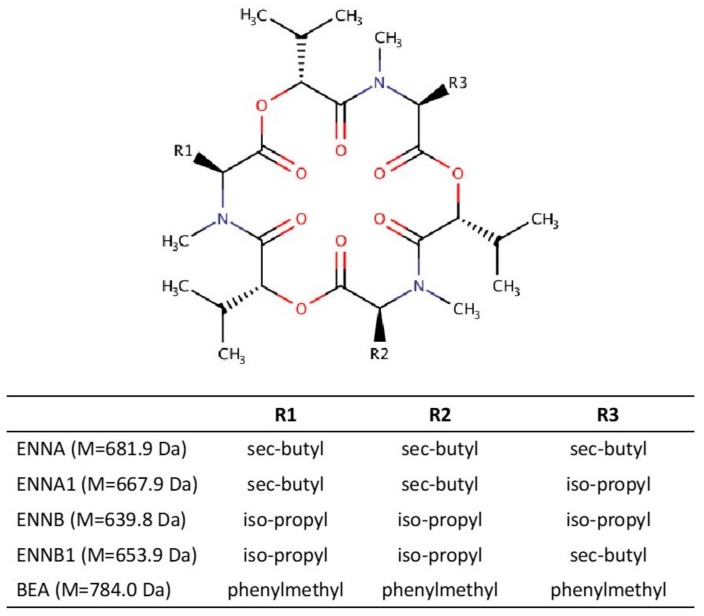
Chemical structures of enniatins and beauvericin.

Enniatins and beauvericins are cyclic hexadepsipeptide compounds. Enniatins are made of six residues that vary between N-methyl amino acids and hydroxylated carboxylic acids, whereas beauvericins have a core structure made up of three N-methyl-L-phenylalanine blocks connected alternately with three 2-hydroxy-D-isovaleric acid units [6]. About 44 enniatins and beauvericins have been isolated and determined, the most commonly found in food and animal feed being enniatins A (ENNA), A1 (ENNA1), B (ENNB), B1 (ENNB1), and beauvericin (BEA) (Figure 1), all of which have been described on a worldwide scale [5,7,8,9,10,11,12]. Some studies have demonstrated the presence of these toxins in 100% of the samples analyzed [11,12]. However, it is difficult to measure real exposure to mycotoxins based on their occurrence described in animal feed and food—an effective alternative might be monitoring them in biological liquids such as urine or blood (serum and plasma). The mycotoxins present in biological fluids could be considered as biomarkers for the consumption of contaminated foodstuff. Among biological fluids, urine is the most often used for measuring toxin exposure [13] since big amounts can be collected simply and non-invasively.

The determination of mycotoxins in biological samples requires sensitive, effective, and accurate methods, as these compounds appear at very low concentration levels. According to EFSA recommendations, the application of liquid chromatography with tandem mass spectrometry (LC-MS/MS) is recommended rather than LC with UV detection, since matrix effects can be better handled, and concentrations below 1 µg/kg can be quantified [5]. 

The determination of ENNA, ENNA1, ENNB, ENNB1, and BEA has been carried out in urine [14,15,16,17,18,19], and serum [14,15,16,18,20,21,22,23,24], from both humans and animals, but also in feces samples [15,16,18] and in different organs and tissues from animals [25,26,27,28]. The vast majority of these analytical methods are based on LC-MS/MS, although LC coupled to high resolution mass spectrometry (HRMS) has also been proposed [17,18,19]. 

Regarding sample treatment, solid-phase extraction (SPE) [14,26,27] and liquid–liquid extraction (LLE) using ethyl acetate or acetonitrile [15,16,18,21,22,23,25,26,27,28] are the most commonly used. Miniaturized techniques like dispersive liquid–liquid microextraction (DLLME) have also been proposed [24] and, recently, Rodríguez-Carrasco et al. [19] compared and evaluated three different sample preparation approaches (dilute and shoot, DLLME, and salting-out liquid–liquid extraction (SALLE)) for the determination of ENNB and its phase I metabolites in human urine samples, concluding that SALLE showed satisfactory validation results. Moreover, Escrivá et al. [17] also compared three extraction methods, SALLE, DLLME, and miniQuEChERS (quick, easy, cheap, effective, rugged, and safe) for the determination of ENNA, ENNA1, ENNB, ENNB1, and BEA together with another six mycotoxins in human urine and, in this case, DLLME was selected as the most suitable methodology.

In recent years, the use of magnetic nanoparticles (MNPs) has increased for sample treatment. More specifically, MNPs can be used as magnetic sorbents in SPE (MSPE) for the separation and preconcentration of different analytes [29]. The magnetic sorbent can also be dispersed in the sample directly (dispersive magnetic solid-phase extraction, DMSPE), instead of using a solid-phase packed cartridge, enhancing the mass transfer and improving the extraction efficiency [30], while reducing the volume of organic solvent needed in the desorption step. In DMSPE, MNPs can be separated simply and rapidly by applying an external magnetic field, avoiding filtration or centrifugation steps. However, despite its multiple advantages, to the best of our knowledge, DMSPE has not been applied for the determination of emerging mycotoxins.

In this study, the potential of DMSPE has been investigated for the determination of ENNA, ENNA1, ENNB, ENNB1, and BEA in human urine samples, and a magnetic multiwalled carbon nanotube (Fe_3_O_4_@MWCNT) composite is applied for the first time as sorbent material for the preconcentration of these mycotoxins before determination by UHPLC-HRMS. 

## 2. Results and Discussion

### 2.1. Optimization of Sample Treatment

In order to get the best extraction conditions, different materials were evaluated for the preparation of the magnetic nanoparticles: β-cyclodextrin (β-CD) [31], polydopamine (PDA) [32], chitosan [33], oleic acid [34], polystyrene (PS) [35], Fe_3_O_4_@MWCNTs [36], MWCNTS/Fe_3_O_4_/polypyrrole (PPy) [36], PPy-nanotubes (NTs) [37] and magnetic cellulose particles [38], and desorption solvents, methanol (MeOH), and acetonitrile (MeCN). For this study, a mass of 20 mg of each nanoparticle type was added to 5 mL of sample spiked at 100 ng/mL before submitting the mixture to orbital shaking for 20 min. Then, 3 mL of extraction solvent (MeOH or MeCN) was used to desorb the mycotoxins from the nanomaterial. A total of 18 experiments were carried out and the best results were obtained with the combination of Fe_3_O_4_@MWCNTs and MeCN. The results are shown in Supplementary Materials (Figure S1).

Once the type of nanoparticle and the composition of the desorption solvent had been selected, the other factors influencing the extraction efficiency in DMSPE were optimized: sample volume, nanoparticle mass, absorption and desorption times, volume of desorption solvent, and percentage of NaCl in the sample. Initially, sample volume was investigated between 5 and 40 mL. The best preconcentration factor was obtained using 30 mL, which was therefore selected as the optimum volume. Then, the mass of nanoparticles, and absorption and desorption times were optimized by means of a multivariate method, to consider potential interactions between the variables. A central composite design (2^3^ + star, face centered), with three spaced central points, including 17 runs, was used to create the response surface, using the peak area as analytical response. The different factors were evaluated in the following ranges: mass of nanoparticles (5–35 mg), absorption time (5–40 min), and desorption time (1–10 min). 

**Figure 2:**
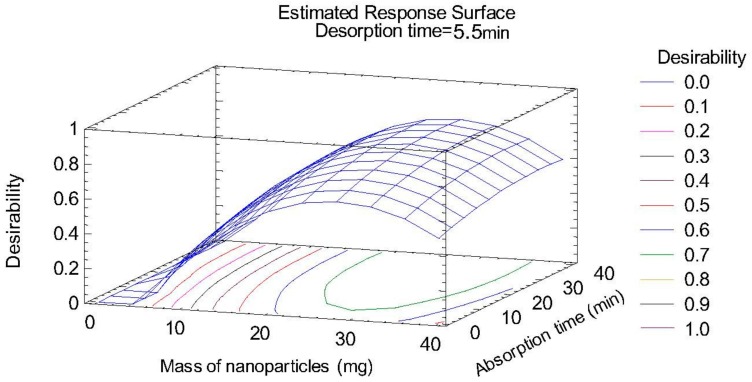
Estimated response surface for the study of significant variables in the dispersive magnetic solid-phase extraction (DMSPE) step obtained by multiple response optimization.

The nanoparticle mass and its quadratic term had a significant effect on the recovery of the target mycotoxins. Subsequently, multiple response optimization was carried out using the desirability function; the response surface plot is shown in Figure 2. The determination coefficient (R^2^) obtained for the target analytes ranged between 83.7% and 92.1%, confirming the suitability of the design. The optimum conditions were as follows: nanoparticles mass, 30 mg; absorption time, 35 min; and desorption time, 5 min.

After the multivariate study, the desorption solvent volume between (0.5 and 5 mL) and percentage of NaCl in the sample between (0 and 10% wt.) were optimized using a univariate method. No significant differences were found for the different salt percentages, and so NaCl was not added to the sample for the DMSPE procedure. In the case of desorption solvent, the best preconcentration and recovery results were obtained using 1.5 mL of MeCN.

Under these optimum conditions, the obtained preconcentration factors were 95, 35, 44, 19, and 34 for ENNB, ENNB1, ENNA1, ENNA, and BEA, respectively. The preconcentration factors were calculated as the ratio of the slope of the calibration curves applying the DMSPE method and the slope of the calibration curves obtained by direct injection of the standards in urine into the UHPLC-HRMS system.

### 2.2. Characterization of Nanocomposite

The characterization of the multiwalled carbon nanotube (MWCNT) and magnetic multiwalled carbon nanotube (Fe_3_O_4_@MWCNT) nanocomposite was carried out by scanning electron microscopy (SEM), energy dispersive X-ray spectrometry (EDS), X-ray diffraction (XRD), and Fourier transform infrared spectrophotometry (FT-IR). 

**Figure 3:**
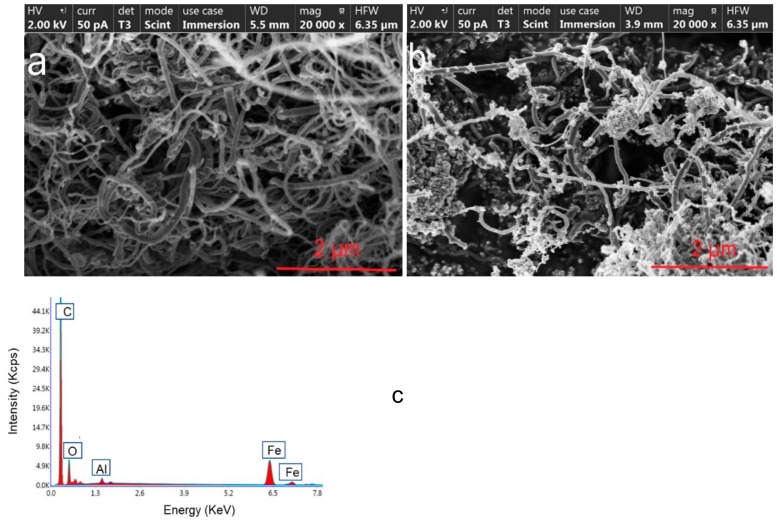
Scanning electron microscopy (SEM) images of multiwalled carbon nanotube (MWCNT) (**a**) and Fe_3_O_4_@MWCNT (**b**) nanocomposite. Energy dispersive X-ray spectrometry (EDS) spectrum of Fe_3_O_4_@MWCNT (**c**).

The pictures obtained by SEM show the morphology of the MWCNT (Figure 3a) and the synthetized Fe_3_O_4_@MWCNT (Figure 3b) material, where it can be appreciated how the iron oxide nanoparticles were fixed on the surface of the MWCNTs to produce Fe_3_O_4_@MWCNTs.

Peaks related to Fe, C, and O atoms were observed in the EDS spectrum of the Fe_3_O_4_@MWCNT adsorbent material (Figure 3c). The quantitative analysis gave weight ratios of 14.95%, 68.06%, and 15.49% for Fe, C, and O, respectively. Aluminum was used as support for the measurements.

**Figure 4:**
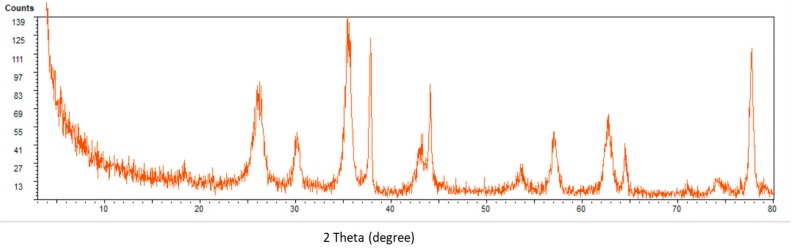
X-ray diffraction (XRD) spectrum of Fe_3_O_4_@MWCNT nanocomposite.

Analysis of the XRD data using XPOWDER software [39] showed the presence of two types of magnetic iron oxide; magnetite (Fe_3_O_4_, around 10% wt.) and maghemite (gamma-Fe_3_O_4_, around 61% wt.). The standard X-ray diffraction peaks (2ϴ = 30.014°, 35.578°, 43.180°, 53.652°, 56.788°, and 62.757°) which can be assigned to maghemite or magnetite match with those observed in the spectrum depicted in Figure 4. The other signals are related to the oxidized and non-magnetized carbon nanotubes (Carbon and Graphite). 

**Figure 5:**
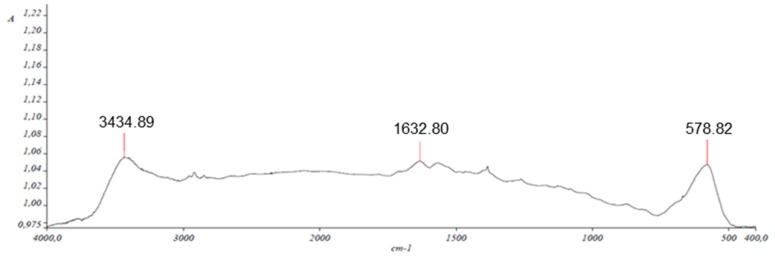
Fourier transform infrared spectrophotometry (FTIR) spectrum of MWCNT nanocomposite.

The Fe_3_O_4_@MWCNT material was also analyzed by FTIR to determine the functional groups present on the surface of the nanocomposite particles. Figure 5 shows several bands related to the carboxylic acid groups formed during the oxidation step of the nanoparticle synthesis process. Note the COO^−^ asymmetric stretching band at 1633 cm^−1^, the 3435 cm^−1^ signal due to the stretching band for O-H, and the 578 cm^−1^ signal for characteristic Fe-O band.

### 2.3. Method Validation

In order to evaluate the suitability of the proposed method for the determination of emergent mycotoxins in urine, an in-depth validation was carried out by evaluating the linear dynamic ranges, limits of detection (LOD) and quantification (LOQ), matrix effect (ME), trueness, and precision. 

The ME (Table 1) was calculated as 100 × [(signal of analyte in sample extract−signal of analyte in neat solvent)/signal of analyte in neat solvent] and was evaluated at three concentration levels for each emergent mycotoxin (0.1, 5, and 25 μg/L). A significant signal suppression effect was found, and matrix-matched calibration curves were therefore necessary for quantification purposes. 

Matrix-matched calibration curves were set up using urine samples spiked at six concentrations levels between 0.1 and 50 μg/L of emergent mycotoxins. Each concentration level was processed in triplicate and also injected in triplicate, considering peak area as the analytical signal. Determination coefficients above 0.99 (Table 1) were obtained in all cases, confirming satisfactory linearity over the whole studied range.

The LODs and LOQs were calculated using the criteria of 3 and 10 times the signal-to-noise ratio (S/N), respectively. Low LOQs were obtained for all the target mycotoxins, which were similar to [14,17] or lower than [15,16,18] those obtained with other analytical methods described in the literature. 

The precision of the method was studied in terms of repeatability (intraday precision) and intermediate precision (interday precision). Repeatability was evaluated by applying the complete procedure to three samples (experimental replicates) spiked at three different concentration levels of each emergent mycotoxin (0.1, 5, and 25 μg/L). All the samples were measured on the same day, and each extract was injected in triplicate (instrumental replicates). Intermediate precision was assessed with a similar procedure, spiking and analyzing four different samples on four different days. The results of the precision study, expressed as the relative standard deviation (RSD, %) of analyte peak area are shown in Table 1 and, in all cases, RSD values lower than 12% were obtained, conforming with the legislation concerning other mycotoxins [40].

In order to evaluate the trueness of the proposed method, recovery experiments were performed with urine samples previously analyzed to establish the lack of detectable mycotoxins. None of them provided a positive result above the LODs of the method. These samples were spiked at three different concentration levels (0.1, 5, and 25 μg/L), treated as described previously and injected in triplicate into the UPLC-HRMS equipment. Recoveries ranged between 89.3% and 98.9%, fulfilling the requirements of current legislation for other mycotoxins [40]. 

### 2.4. Application to Urine Samples

The suitability of the method was finally evaluated by analyzing ten samples of human urine according to the optimized method. ENNA, ENNA1, ENNB, ENNB1, and BEA were not detected in any of the samples.

### 2.5. Nanomaterial Reuse

**Figure 6:**
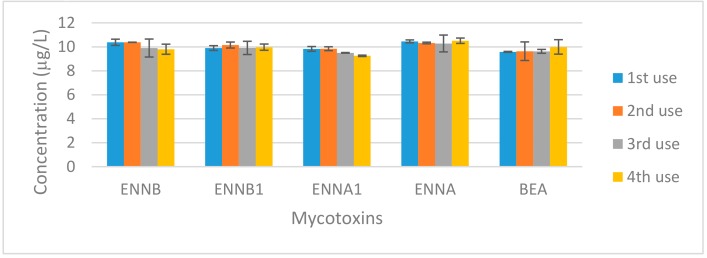
Fe_3_O_4_@MWCNTs reuse study.

One of the disadvantages of DMSPE is that it requires a stage of magnetic material synthesis, which must be carried out very carefully to obtain reproducible results, thereby slowing down the analytical procedure. One way to avoid this disadvantage would be the possibility of reusing the nanomaterial. For this reason, a study of the reuse of the Fe_3_O_4_@MWCNT composite synthetized for the determination of emergent mycotoxins in urine was carried out. For the assay, 30 mg of Fe_3_O_4_@MWCNTs was used to extract the five mycotoxins at 10 μg/L from 30 mL of urine and, after a desorption step, the nanocomposite was used to consecutively extract another three urine samples fortified at the same concentration level. Although a small loss of nanoparticle mass was assumed during the sample treatment, the results, shown in Figure 6, confirmed that it can be reused at least 4 times. The only significant difference in concentration was observed for ENNA1, where a decrease in the signal of approximately 6% was observed.

## 3. Conclusions

In this work, the applicability of MNPs in sample treatment for the determination of emergent mycotoxins (ENNA, ENNA1, ENNB, ENNB1, and BEA) before their determination by UHPLC-HRMS has been demonstrated for the first time. The proposed method based on DMSPE has multiple advantages over other sample treatments, such as the enhancement of the mass transfer, the improvement of the extraction efficiency, and the reduction of organic volume solvent in the desorption step, avoiding filtration or centrifugation steps. A wide variety of the most used nanoparticles for other types of contaminants were explored (β-CD, PDA, chitosan, oleic acid, PS, Fe_3_O_4_@MWCNTs, MWCNTS/Fe_3_O_4_/PPy, PPy-NTs, and magnetic cellulose particles) and Fe_3_O_4_@MWCNTs showed the best result in term of recovery. The proposed methodology was applied for the determination of the target mycotoxins in urine samples since the determination of mycotoxins in biological samples requires sensitive, effective, and accurate methods, as these compounds appear at very low concentration levels. The DMSPE-UHPLC-HRMS method allowed to achieve low LOD and LOQ, enabling the detection of target mycotoxins at levels normally found in urine. In addition, the precision was lower than 12% and recoveries ranged between 89.3% and 98.9%, demonstrating that the Fe_3_O_4_@MWCNT composite can be reused at least four times. 

## 4. Materials and Methods 

### 4.1. Chemicals, Reagents, and Standards

Individual standards of ENNA, ENNA1, ENNB, ENNB1, and BEA were obtained from Sigma Aldrich (St. Louis, MO, USA). Mycotoxin stock solutions were prepared at 1 mg/L in acetonitrile (MeCN) and stored at −20 °C. Ethanol and MeCN were provided by ChemLab (Zedelgem, Belgium). Nitric acid (65%) was purchased from Panreac (Barcelona, Spain). Iron chloride (III) (>99%) (FeCl_3_), ammonium iron (II) sulfate hexahydrate ((NH_4_)_2_Fe(SO_4_)_2_⋅6H_2_O), ammonia solution, and sodium chloride were also purchased from Sigma-Aldrich. MeOH and formic acid (HCOOH) used as mobile phase were LC-MS grade and were also obtained from Sigma-Aldrich.

MWCNTs (average diameters between 40 and 60 nm, average length of >5 μm, specific surface area of 40–70 m^2^/g) were provided by Shenzhen Nanotech Port Co., Ltd. (Shenzhen, China). For the filtration of samples before the chromatographic analysis, nylon syringe filters, 0.22 µm × 25 mm (Agela Technologies, New York, NY, USA) were used.

### 4.2. Instrumentation and Software

The UHPLC system consisted of an Agilent 1290 Infinity II Series HPLC (Agilent Technologies, Santa Clara, CA, USA) provided with an automated multisampler unit and a high speed binary pump, coupled to an Agilent 6550 Q-TOF mass spectrometer (Agilent Technologies, Santa Clara, CA, USA) equipped with an ESI source (Agilent Jet Stream Dual electrospray, AJS-Dual ESI, Santa Clara, CA, USA). For data processing, MassHunter Workstation Data Acquisition software (Agilent Technologies, Rev. B.08.00, Santa Clara, CA, USA) was used. The statistic software Statgraphics Centurion XV.II was used for data treatment.

A Unicen-21 centrifuge (TQTech, Shenzhen, China), an IKA-KS-130-Basic orbital agitator (IKA Werke GmbH & Co KG, Staufen, Germany), a vortex stirrer LLG-uniTEXER (Serviquimia, Constantí, Tarragona), a rotavapor (BUCHI, Labortechnink:AG: Flawil, Switzerland), and an Xcelvap air-drying system (Horizon Technology Inc., Salem, NH, USA) were also used for sample treatment. 

Permanent magnets were purchased from Supermagnete (Gottmadingen, Germany). The magnets were blocks composed of Nd-Fe-B (50 × 15 × 15 mm and 86 g weight) with a strength of 33 kg. 

### 4.3. Synthesis of Fe3O4@MWCNTs Composite

The magnetic Fe_3_O_4_@MWCNT composite material was synthetized following the methodology described by Asgharinezhad and Ebrahimzadeh [36]. In a first step, MWCNTs were purified with 1 M nitric acid solution for 6 h at room temperature and then washed many times with distilled water and dried in an oven at 100 °C. Then, about 0.5 g of the purified nanotubes were added into 250 mL of a solution containing 0.85 g (NH_4_)_2_Fe(SO_4_)_2_⋅6H_2_O and 0.4222 g FeCl_3_ at 50 °C. After that, the suspension was sonicated for 20 min and 20 mL of 8 M ammonia solution was added dropwise to precipitate the magnetized particles while the solution was still under sonication. The pH was controlled to ensure that it remained in the 10–11 range by adding ammonia solution 25% (*w*/*w*). In order to enhance the whole growth of the nanoparticle crystals, the reaction was allowed to proceed at 50 °C for 30 min. The dispersion was cooled to room temperature, the Fe_3_O_4_@MWCNT were collected by a strong permanent magnet and washed three times with deionized water followed by ethanol. The magnetic composite substance was dried at 60 °C overnight and lastly ground in a mortar and kept at room temperature in an amber glass vial.

### 4.4. Sample Preparation

Urine samples were obtained from healthy volunteers and were collected in sterile plastic containers and refrigerated until analysis. For sample treatment, 30 mg of Fe_3_O_4_@MWCNTs and 30 mL of urine were placed in a test tube, which was orbitally shaken for 35 min at room temperature. The nanocomposite was then attracted with the external neodymium magnet and the supernatant solution was discarded. 

In order to desorb the emergent mycotoxins, 1.5 mL of MeCN was added and the mixture was shaken using the orbital shaker for 5 min at room temperature. After that, the nanomaterial was again attracted with the magnet and in this case, the supernatant solution was recovered. A 250 µL volume of the recovered organic phase was mixed with 250 µL of water and filtered through a 0.2 mm filter before injection in the UHPLC-Q-TOF system.

### 4.5. UHPLC-HRMS Analysis

The separation and determination of emergent mycotoxins was performed in a ZORBAX RRHD Eclipse Plus C18 (1.8 μm, 2.1 × 100 mm) column equipped with a 0.3 μm inline filter from Agilent Technologies and using a mobile phase consisting of H_2_O:MeOH (95:5, *v*/*v*) containing 0.1% HCOOH (solvent A) and MeOH:H_2_O (95:5, *v*/*v*) containing 0.1% HCOOH (solvent B) at a flow rate of 0.4 mL/min. The elution program was applied as follows: 0–1 min: 70% B; 1–3 min: 70–100% B; 3–5 min: 100% B; 5–5.2 min: 100–70% B; 5.2–9 min: 70% B. The column temperature was set at 35 °C and the autosampler temperature was 5 °C. A volume of 20 μL of the sample was injected.

The mass spectrometer worked in the positive mode. The nebulizer gas pressure was established to 30 psi, while the drying gas flow was set to 16 L/min at a temperature of 130 °C, and the sheath gas flow was set to 11 L/min at a temperature of 300 °C. The capillary spray, nozzle, fragmentor, and 1 RF Vpp octopole voltages were 4000, 500, 360, and 750 V, respectively. Data acquisition was carried out using “All-ion mode” and three collision energies (0, 10, and 40 V) were measured. Profile data in the 50–1500 *m/z* range were acquired for MS scans in 2 GHz extended dynamic range mode with 3 spectra/s, 333.3 ms/spectrum, and 2675 transients/spectrum. Reference masses of 121.0509 and 922.0098 *m/z* were used for mass correction during the analysis. For quantification purposes, extracted ion chromatograms (EIC) from the full-scan MS data were obtained for the protonated molecule of each analyte with a 5 ppm window. The monitored ions of the target analytes are shown in Table 2, as well as their retention times, the theoretical and experimental *m/z* values, and the corresponding instrumental error, which was calculated (in terms of percentage) as the difference between the experimental and theoretical *m/z* values divided by the theoretical *m/z* value multiplied by 10^6^.

**Figure 7:**
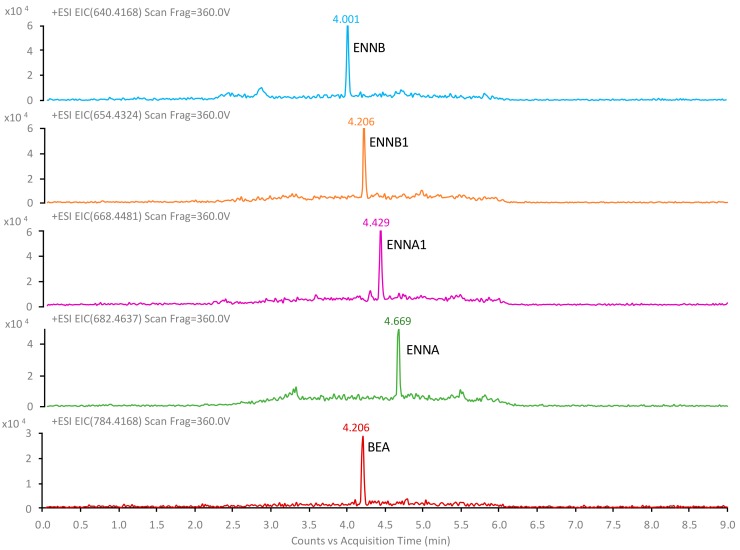
UHPLC–HRMS extracted ion chromatograms of a spiked urine sample at 0.1 μg/L analyzed with the proposed method.

Figure 7 shows a chromatogram obtained with the method proposed for the determination of emergent mycotoxins in urine.

## Figures and Tables

**Table 1:** Method validation data for the determination of emergent mycotoxins in urine.

Mycotoxin	Equation		Linear Range (μg/L)	Linearity R^2^	LOD (μg/L)	LOQ (μg/L)
ENNA	y = 629312 x + 871432		0.04–50	0.996	0.01	0.04
ENNA1	y = 1755799 x + 1381024		0.10–50	0.995	0.03	0.10
ENNB	y = 833213 x + 669371		0.04–50	0.995	0.01	0.04
ENNB1	y = 1883668 x + 3420346		0.04–50	0.992	0.01	0.04
BEA	y = 1468318 x – 1256332		0.04–50	0.993	0.01	0.04
	**Matrix Effect (%)**			**Trueness (%)**		
	**0.1 μg/L**	**5 μg/L**	**25 μg/L**	**0.1 μg/L**	**5 μg/L**	**25 μg/L**
ENNA	–12.5	–18.6	–17.0	97.0	92.2	89.3
ENNA1	–33.6	–34.3	–22.3	96.9	92.4	93.9
ENNB	–33.7	–37.8	–37.2	97.6	95.7	98.9
ENNB1	–5.1	–8.7	–12.1	98.6	98.3	98.0
BEA	–35.5	–20.3	–35.1	96.7	90.5	94.4
	**Repeatability, %RSD (*n* = 9)**			**Intermediate Precision, %RSD (*n* = 12)**		
	**0.1 μg/L**	**5 μg/L**	**25 μg/L**	**0.1 μg/L**	**5 μg/L**	**25 μg/L**
ENNA	6.0	6.7	6.9	10.1	8.2	7.4
ENNA1	6.8	8.0	8.6	7.5	8.5	10.2
ENNB	8.9	9.4	8.7	10.2	9.3	9.9
ENNB1	8.5	6.9	5.9	8.6	9.1	11.7
BEA	8.7	8.8	9.0	8.9	9.0	9.1

**Table 2:** Monitored ions of the target analytes.

Compound	t_R_ (min)	Formula	*m/z* Theoretical	*m/z* Experimental	Error (ppm)	Q1, *m/z*	Q2, *m/z*
ENNB	4.00	C_33_H_58_N_3_O_9_^+^	640.4168	640.4173	0.8	196.1341	214.1441
ENNB1	4.21	C_34_H_60_N_3_O_9_^+^	654.4324	654.4326	0.3	196.1333	210.1489
BEA	4.21	C_45_H_58_N_3_O_9_^+^	784.4168	784.4163	−0.6	244.1334	262.1438
ENNA1	4.43	C_35_H_62_N_3_O_9_^+^	668.4481	668.4485	0.6	210.1491	228.1592
ENNA	4.67	C_36_H_64_N_3_O_9_^+^	682.4637	682.4636	−0.1	210.1491	228.1593

## Supplementary Materials

The following are available online at https://www.mdpi.com/2072-6651/12/1/51/s1, Figure S1: Study of different materials for the preparation of the magnetic nanoparticles and desorption solvents (MeCN (**a**) and MeOH (**b**)) for the extraction of emergent mycotoxins (*n* = 3).

## References

[1] Commission of the European Communities (2006). Regulation (EC) No. 1881/2006 setting maximum levels for certain contaminants in foodstuffs. Off. J. Eur. Commun..

[2] European Commission (2012). Commission recommendation No. 2012/154/UE on the monitoring of the presence of ergot alkaloids in feed and food. Off. J. Eur. Commun..

[3] Commission of the European Communities (2013). Commission recommendation No. 2013/165/EU on the presence of T-2 and HT-2 toxin in cereals and cereal products. Off. J. Eur. Commun..

[4] Mamur S., Yuzbasioglu D., Yılmaz S., Erikel E., Unal F. (2018). Assessment of cytotoxic and genotoxic effects of enniatin-A *in vitro*. Food Addit. Contam. Part A.

[5] EFSA Panel on Contaminants in the Food Chain (CONTAM) (2014). Scientific opinion on the risks to human and animal health related to the presence of beauvericin and enniatins in food and feed. EFSA J..

[6] Wang X., Gong X., Li P., Lai D., Zhou L. (2018). Structural diversity and biological activities of cyclic depsipeptides from fungi. Molecules.

[7] Juan C., Mañes J., Raiola A., Ritieni A. (2013). Evaluation of beauvericin and enniatins in Italian cereal products and multicereal food by liquid chromatography coupled to triple quadrupole mass spectrometry. Food Chem..

[8] Juan C., Ritieni A., Mañes J. (2013). Occurrence of Fusarium mycotoxins in Italian cereal and cereal products from organic farming. Food Chem.

[9] Kim D.-B., Song N.-E., Nam T.G., Lee S., Seo D., Yoo M. (2019). Occurrence of emerging mycotoxins in cereals and cereal-based products from the Korean market using LC-MS/MS. Food Addit. Contam. Part A.

[10] Han X., Xu W., Zhang J., Xu J., Li F. (2019). Natural occurrence of beauvericin and enniatins in corn-and wheat-based samples harvested in 2017 collected from Shandong province, China. Toxins.

[11] Arroyo-Manzanares N., Rodríguez-Estévez V., Arenas-Fernández P., García-Campaña A.M., Gámiz-Gracia L. (2019). Occurrence of mycotoxins in swine feeding from Spain. Toxins.

[12] Uhlig S., Torp M., Heier B.T. (2006). Beauvericin and enniatins A, A1, B and B1 in Norwegian grain: A survey. Food Chem..

[13] Huybrechts B., Martins J.C., Debongnie P., Uhlig S., Callebau A. (2015). Fast and sensitive LC–MS/MS method measuring human mycotoxin exposure using biomarkers in urine. Arch. Toxicol..

[14] Serrano A.B., Capriotti A.L., Cavaliere C., Piovesana S., Samperi R., Ventura S., Laganà A. (2015). Development of a rapid LC-MS/MS method for the determination of emerging fusarium mycotoxins enniatins and beauvericin in human biological fluids. Toxins.

[15] Juan C., Manyes L., Font G., Juan-Garcia A. (2014). Evaluation of immunologic effect of Enniatin A and quantitative determination in feces, urine and serum on treated Wistar rats. Toxicon.

[16] Escrivá L., Font G., Manyes L. (2015). Quantitation of enniatins in biological samples of Wistar rats after oral administration by LC-MS/MS. Toxicol Mech. Methods.

[17] Escrivá L., Manyes L., Font G., Berrada H. (2017). Mycotoxin analysis of human urine by LC-MS/MS: A comparative extraction study. Toxins.

[18] Lauwers M., De Baere S., Letor B., Rychlik M., Croubels S., Devreese M. (2019). Multi LC-MS/MS and LC-HRMS methods for determination of 24 mycotoxins including major phase I and II biomarker metabolites in biological matrices from pigs and broiler chickens. Toxins.

[19] Rodríguez-Carrasco Y., Izzo L., Gaspari A., Graziani G., Mañesa J., Ritieni A. (2018). Urinary levels of enniatin B and its phase I metabolites: First human pilot T biomonitoring study. Food Chem. Toxicol..

[20] Taevernier L., Bracke N., Veryser L., Wynendaele E., Gevaert B., Peremans K., De Spiegeleer B. (2016). Blood-brain barrier transport kinetics of the cyclic depsipeptide mycotoxins beauvericin and enniatins. Toxicol. Lett..

[21] Fraeyman S., Devreese M., Antonissen G., De Baere S., Rychlik M., Croubels S. (2016). Comparative Oral Bioavailability, Toxicokinetics, and Biotransformation of Enniatin B1 and Enniatin B in Broiler Chickens. J. Agric. Food Chem..

[22] Devreese M., De Baere S., De Backer P., Croubels S. (2013). Quantitative determination of the Fusarium mycotoxins beauvericin, enniatin A, A1, B and B1 in pig plasma using high performance liquid chromatography–tandem mass spectrometry. Talanta.

[23] Devreese M., Broekaert N., De Mil T., Fraeyman S., De Backer P., Croubels S. (2014). Pilot toxicokinetic study and absolute oral bioavailability of the Fusarium mycotoxin enniatin B1 in pigs. Food Chem. Toxicol..

[24] Tolosa J., Font G., Mañes J., Ferrer E. (2016). Multimycotoxin analysis in water and fish plasma by liquid chromatography-tandem mass spectrometry. Chemosphere.

[25] Manyes L., Escriva L., BelenSerrano A., Rodriguez-Carrasco Y., Tolosa J., Meca G., Font G. (2014). A preliminary study in Wistar rats with enniatin A contaminated feed. Toxicol. Mech. Methods.

[26] Kongkapan J., Giorgi M., Poapolathep S., Isariyodom S., Poapolathep A. (2016). Toxic kinetics and tissue distribution of nivalenol in broiler chickens. Toxicon.

[27] Tolosa J., Font G., Mañes J., Ferrer E. (2014). Natural occurrence of emerging Fusarium mycotoxins in feed and fish from aquaculture. J. Agric. Food Chem..

[28] Rodríguez-Carrasco Y., Heilos D., Richter L., Süssmuth R.D., Heffeter P., Sulyok M., Kenner L., Berger W., Dornetshuber-Fleiss R. (2016). Mouse tissue distribution and persistence of the food-born fusariotoxins Enniatin B and Beauvericin. Toxicol. Lett..

[29] Herrero-Latorre C., Barciela-García J., García-Martín S., Peña-Crecente R.M., Otárola-Jiménez J. (2015). Magnetic solid-phase extraction using carbon nanotubes as sorbents: A review. Anal. Chim. Acta.

[30] Jing W., Zhou Y., Wang J., Ni M., Bi W., Chen D.D.Y. (2019). Dispersive Magnetic Solid-Phase Extraction Coupled to Direct Analysis in Real Time Mass Spectrometry for High-Throughput analysis of trace environmental contaminants. Anal. Chem..

[31] Zhang X., Wang Y., Yang S. (2014). Simultaneous removal of Co (II) and 1-naphthol by core-shell structured Fe_3_O_4_@cyclodextrin magnetic nanoparticles. Carbohydr. Polym..

[32] Huang Z., Lee H.K. (2015). Study and comparison of polydopamine and its derived carbon decorated nanoparticles in the magnetic solid-phase extraction of estrogens. J. Chromatogr. A.

[33] Gopal J., Abdelhamid H.N., Hua P.Y., Wu H.F. (2013). Chitosan nanomagnets for effective extraction and sensitive mass spectrometric detection of pathogenic bacterial endotoxin from human urine. J. Mater. Chem..

[34] Benedé J.L., Chisvert A., Giokas D.L., Salvador A. (2014). Development of stir bar sorptive-dispersive microextraction mediated by magnetic nanoparticles and its analytical application to the determination of hydrophobic organic compounds in aqueous media. J. Chromatogr. A.

[35] Yu X., Sun Y., Jiang C., Sun X., Gao Y., Wang Y., Zhang H., Son D. (2012). Magnetic solid-phase extraction of five pyrethroids from environmental water samples followed by ultrafast liquid chromatography analysis. Talanta.

[36] Asgharinezhad A.A., Ebrahimzadeh H. (2015). Coextraction of acidic, basic and amphiprotic pollutants using multiwalled carbon nanotubes/magnetite nanoparticles@polypyrrole composite. J. Chromatogr. A.

[37] Upadhyay J., Kumar A., Gogoi B., Buragohain A.K. (2015). Antibacterial and hemolysis activity of polypyrrole nanotubes decorated with silver nanoparticles by an in-situ reduction process. Mater. Sci. Eng. C.

[38] Periyasamy S., Gopalakannan V., Viswanathan N. (2017). Fabrication of magnetic particles imprinted cellulose based biocomposites for chromium (VI) removal. Carbohyd. Polym..

[39] Martin J.D. (2006). XPowder. www.xpowder.com.

[40] Commission of the European Communities (2006). Regulation (EC) No. 401/2006 of laying down the methods of sampling and analysis for the official control of the levels of mycotoxins in foodstuffs. Off. J. Eur. Commun..

