# Supplementary material for: Dispersive Solid-Phase Extraction Using Magnetic Carbon Nanotube Composite for the Determination of Emergent Mycotoxins in Urine Samples
Source: Toxins (Basel). 2020 Jan 15;12(1):51. doi: 10.3390/toxins12010051 (PMC7020456; doi:10.3390/toxins12010051)
Supplement: Supplementary file 1 [file toxins-12-00051-s001.pdf]

Supplementary Materials: Dispersive Solid-Phase Extraction using Magnetic Carbon Nanotube Composite for the Determination of Emergent Mycotoxins in Urine Samples

Natalia Arroyo-Manzanares, Rosa Peñalver-Soler, Natalia Campillo and Pilar Viñas

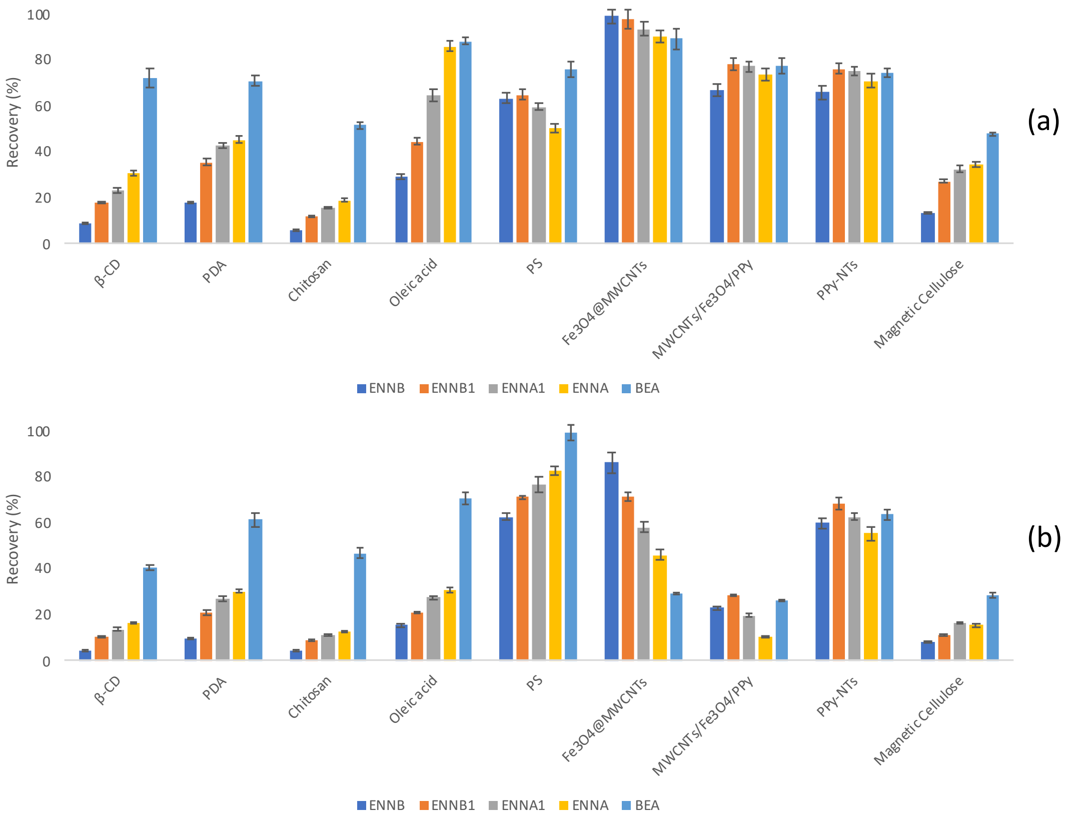

Figure S1. Study of different materials for the preparation of the magnetic nanoparticles and desorption solvents (MeCN (**a**) and MeOH (**b**)) for the extraction of emergent mycotoxins ($n = 3$).
